# Hierarchically Porous Polyacetylene Networks: Adsorptive Photocatalysts for Efficient Bisphenol A Removal from Water

**DOI:** 10.1021/acspolymersau.4c00032

**Published:** 2024-06-06

**Authors:** David Šorm, Jiří Brus, Albin Pintar, Jan Sedláček, Sebastijan Kovačič

**Affiliations:** † Department of Physical and Macromolecular Chemistry, Faculty of Science, Charles University, Hlavova 2030, Prague 2 128 43, Czech Republic; ‡ Institute of Macromolecular Chemistry, Czech Academy of Sciences, Heyrovský Sq. 2, 162 00 Prague, Czech Republic; § Department of Inorganic Chemistry and Technology, National Institute of Chemistry, Hajdrihova 19, SI-1001 Ljubljana, Slovenia; ∥ Faculty of Chemistry and Chemical Engineering, University of Maribor, Smetanova 17, SI-2000 Maribor, Slovenia

**Keywords:** polyacetylenes, emulsion-templating, π-conjugated networks, macroporous polymers, heterogeneous photocatalysis

## Abstract

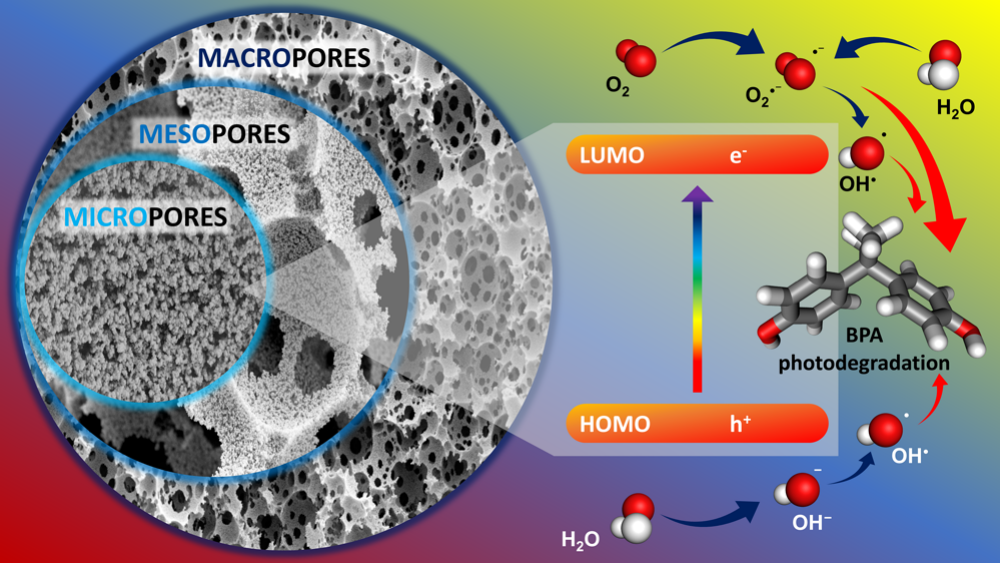

In this article, we report a series of functionalized polyacetylene-type networks formed by chain-growth insertion coordination polymerization in high internal phase emulsions (HIPEs). All polymerized HIPEs (polyHIPEs) contain a hierarchically structured, 3D-interconnected porous framework consisting of a micro-, meso- and macropore system, resulting in exceptionally high specific surface areas (up to 1055 m^2^·g^–1^) and total porosities of over 95%. The combination of π-conjugated and hierarchically porous structure in one material enabled the use of these polyacetylene polyHIPEs as adsorptive photocatalysts for the removal of chemical contaminants from water. All polyacetylene polyHIPEs demonstrated high efficiency in the adsorption of bisphenol A from water (up to 48%) and the subsequent photocatalytic degradation. Surprisingly, high adsorption capacity did not affect the photocatalytic efficiency (up to 58%). On the contrary, this dual function seems to be very promising, as some polyacetylene polyHIPEs almost completely removed bisphenol A from water (97%) through the adsorption-photooxidation mechanism. It also appears that the presence of polar functional side groups in the polyacetylene backbone improves the contact of the polyacetylene network with the aqueous bisphenol A solution, which can thus be more easily adsorbed and subsequently oxidized, compensating for the lower specific surface area of some networks, namely, 471 and 308 m^2^·g^–1^ in the case of 3-ethynylphenol- and 3-ethynylaniline-based polyacetylene polyHIPEs, respectively.

## Introduction

Synthetic chemical contaminants such as per- and polyfluoroalkyl substances (PFAS) or bisphenol A (BPA) and its analogues, to name a few, are persistent, bioaccumulative pollutants found in water resources in low concentrations but with a significant adverse effect on human health.^1^ Unless appropriate actions are taken regarding chemical water pollution at both the technological and societal levels, it is estimated that by 2050, more than half of the world’s population will be affected by water stress—a situation where demand exceeds the available amount of good quality water.^2^

Considering the environmental persistence, toxicity, and bioaccumulation of synthetic organic contaminants, numerous efforts have been made to remove them from water bodies, including adsorption, filtration, reverse osmosis, enhanced photolysis, electrochemical oxidation, sonochemical destruction, etc.^3^ However, many of these methods are expensive or have low removal efficiency due to high energy requirements. The adsorption process remains one of the most cost-effective and environmentally friendly methods. Compared to conventional sorbents, e.g., ion exchange resins, porous material-based adsorbents with a large surface area, large pore volume, and suitable functional groups on the pore surface are the key to success.^4,5^ Among them, cryogels have attracted great interest in many studies due to their unique porous structure, high adsorption capacity, and ease of handling, which are valuable properties for various applications, especially for water treatment.^6−9^ Another group of porous material-based adsorbents are conjugated porous polymer networks (CPPNs)^10^ appear to be very successful in the removal of persistent organic contaminants such as PFAS or bisphenols.^11−13^ CPPNs combine attractive properties such as high porosity, large surface area, high stability, and extensive π-conjugation. Since they are predominantly built up using aromatic, rigid monomers as building blocks, their polyarylene-based networks have a sufficient fluorous and hydrophobic affinity, which enables an efficient adsorption capacity, e.g., PFAS or bisphenols, respectively, through the large surface area.^14,15^ In recent years, however, the photocatalytic degradation of water-dissolved synthetic organic contaminants has attracted much attention due to the use of light as a renewable, readily available, and sustainable energy source.^16^ In this view, the large π-electron-delocalization of CPPNs is considered key.^17^ It enables efficient light absorption and charge carrier transportation, which is advantageous, e.g., for visible-light-driven photocatalysis.^18^ When CPPNs are used as photocatalysts, one or more reactive oxygen species, i.e., superoxide radicals (O_2_^•–^), hydroxyl radicals (OH^•^), singlet oxygen (^1^O_2_), peroxides (H_2_O_2_), and photogenerated holes (h^+^), are involved in the photooxidation of synthetic organic contaminants.^19^ Benefiting from the highly porous and π-electron-delocalized poly(arylene) network structure, CPPNs could therefore be used both as efficient adsorbents for organic contaminants and as heterogeneous photocatalysts for their degradation.^20^ This dual function seems to be very promising and offers unprecedented advantages for the treatment of organic contaminants in water. Therefore, it is of utmost interest to design and synthesize novel CPPNs that combine a semiconducting and hierarchically porous framework with various functional groups on the pore surface that facilitate the access of water-dissolved organic contaminants to the interior of the CPPN.

Indeed, conjugated polymerized high internal phase emulsions (HIPEs), referred to as “π-conjugated polyHIPEs”,^21,22^ are a unique subclass that differs from other CPPNs by having additional porosity on a larger length scale (pore sizes between 1 and 100 μm). PolyHIPEs (PHs) are typically formed by the polymerization of the external (monomeric) phase of HIPEs, resulting in monolithic polymeric materials with unique three-dimensional (3D)-interconnected microcellular morphology.^23−25^ Originally, the development of PHs was driven by absorbent applications, in particular the absorption of body fluids,^26,27^ and later the adsorption of contaminants from water.^28−31^ Recently, however, it has been shown that PHs can be advantageously used as heterogeneous photocatalysts in singlet oxygen generation,^32^ organic photoredox reactions,^33,34^ photocatalytic sulfoxidation,^35^ or as photoinitiators in radical polymerizations.^36^ In addition, PHs are also able to photodegrade organic contaminants dissolved in water.^37−39^

Polyacetylene (PA) and its derivatives, [–HC=CR–]*_n_* and [–^1^RC=CR^2^–]*_n_*, are probably the first described well-defined π-conjugated polymers.^40^ The π-conjugated nature of these polymers is due to the alternation of single and double bonds between the carbon atoms of the polyene (polyacetylene) main chains. Polyacetylenes are prepared by chain-growth coordination polymerization of acetylene monomers catalyzed by transition-metal complexes operating in metathesis or insertion polymerization mode.^41^ The most frequently used Rh(I) insertion catalysts cleave one π bond of the ethynyl group of the monomer, thereby transforming it into an ethenylene group of the monomeric unit incorporated through propagation into a polyene chain.^42−45^ The Rh(I) catalysts are highly substrate-selective: they transform only ethynyl groups and, contrary to the metathesis catalysts, do not interact with ethenyl groups. Moreover, the Rh(I) catalysts are well compatible with various heteroatom groups of the components of the polymerization systems and operate well also in the presence of water.^46^ As shown earlier, the Rh(I)-catalyzed chain-growth coordination polymerization of acetylene monomers with a higher number of ethynyl groups per molecule leads to the cross-linking of polyacetylene chains and, in optimal cases, also to the formation of a microporous texture of the resulting networks.^47,48^

Herein, we present a simple synthetic strategy that combines high internal phase emulsion (HIPE) templates and rhodium-catalyzed chain-growth coordination polymerization to prepare a series of functionalized conjugated polyacetylene (PA)-based PHs. The rational design of the hierarchically porous framework and the functionalization of the network by incorporating hydrophilic heteroatomic groups enable us the tuning of adsorption capacities and photocatalytic performance of these novel PA–PHs. These bifunctional, i.e., adsorptive and photocatalytically active polymers were then successfully used in the efficient removal of bisphenol A from water.

## Experimental Methods

### Materials

The following compounds were used as received: acetylacetonate(norbornadiene)rhodium(I) [Rh(nbd)acac] (>98%), 1,3-diethynylbenzene (>96%), 1,3,5-triethynylbenzene (>98%), 3-ethynylphenol (>98%), 3-ethynylaniline (>98%) (all TCI Europe), Span80 (sorbitan monooleate, MW = 428 g·mol^–1^, Merck Life Science), calcium chloride dihydrate (99%, Merck Life Science), toluene for analysis (Merck), tetrahydrofuran (≥99%, Sigma-Aldrich), bisphenol A (BPA, *c*_0_ = 10 mg·L^–1^, 100 mL, Aldrich). *N*-Salicylidene(3-ethynylaniline) was prepared according to ref (49) from 3-ethynylaniline and salicylaldehyde (≥99%, Merck Life Science).

### Synthesis of PA–PH Networks

(Co)monomer(s) was/were dissolved in dry toluene (*c*_monomer_ = 1.7 mol·dm^–3^). Surfactant Span80 (sorbitan monooleate, MW = 428 g·mol^–1^) was added to the solution of (co)monomer(s) and properly stirred on the magnetic stirrer. The amount of the surfactant was 15 wt % of the whole solution of (co)monomer(s), including the weight of the solvent. Solution of 1 wt % CaCl_2_ in H_2_O was added dropwise into the solution of (co)monomer(s) (for amount, see the Result and Discussion section) accompanied by intensive stirring. The reaction mixture was stirred for another 20 min to form a uniform emulsion. After that, the stirrer bar was removed and the solution of polymerization initiator [Rh(nbd)acac] in toluene was added to the emulsion. The emulsion was immediately smoothly shaken and the whole reaction mixture was put into the oven at 75 °C for 48 h. During this period, the solid polymer network occurred as a monolith. It should be noted that the HIPEs stopped flowing within minutes of adding the initiator, indicating that gelation occurred very quickly. The gelation was followed by using the vial inversion method. The monolith was isolated and washed in tetrahydrofuran by diffusion for 48 h with frequent exchanges of the solvent. In the end, the product was dried on air at room temperature.

### Adsorption of Bisphenol A

The BPA adsorption was carried out in a batch slurry reactor (Lenz, Wertheim, Germany, model LF60, 250 mL) equipped with a heating/cooling jacket. PA–PH samples were put in contact with 100 mL of aqueous solution, containing the known concentration of BPA (*c*_0_ = 10 mg·L^–1^) wherein 12.5 mg of PA–PH pieces were suspended. The vessel content was thermostated (Julabo F25/ME) at a selected constant temperature (15 °C) with intermittent mixing until equilibration (∼16 h). The BPA concentration in the supernatant was determined with an HPLC instrument (Thermo Scientific, Waltham, MA, model Spectra). The BPA concentration as a function of time was followed at the characteristic BPA wavelength (λ = 210 nm).

### Photocatalytic Degradation of Bisphenol A

Photocatalytic experiments were performed in a batch slurry reactor (Lenz, Wertheim, Germany, model LF60, 250 mL). In all runs, an aqueous solution (ultrapure water, 18.2 MΩ·cm) of bisphenol A (BPA, *c*_0_ = 10 mg·L^–1^, Aldrich) was used. The concentration of the added catalyst was 125 mg·L^–1^. In the middle of the batch slurry reactor, a water-cooled quartz jacket with a visible lamp (Philips 150 W halogen lamp, λ_max_ = 520 nm) was immersed vertically. This enabled us to completely illuminate the BPA solution. To ensure that the catalyst was illuminated only by visible light, a UV cutoff filter at λ = 410 nm from Rosco (E-Color #226: U.V. filter) was used. The degradation of BPA was analyzed with an HPLC instrument (Thermo Scientific, Waltham, MA, model Spectra). The chemical robustness of PA-TEB network was investigated, and the mm-sized pieces were suspended in water purged with air and illuminated with a visible lamp for 24 h. The polymer pieces were then filtered and dried. The water was analyzed by HPLC, while the polymer pieces were subjected to ^13^C CP/MAS NMR.

The amount of BPA adsorbed or oxidized was expressed as the removal percentage and calculated by the equation:
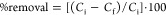
where C_i_ and C_f_ are the initial and final concentrations of contaminants, respectively.

### Methods of Characterization

The ^13^C CP/MAS spectra were recorded at 16.4 T using a Bruker Avance NEO 700 SB NMR spectrometer (Karlsruhe, Germany, 2021) with a 3.2 mm probe head. The MAS frequency was set to 18–20 kHz. The cross-polarization contact time was usually 2 ms, and the dipolar decoupling SPINAL64 was applied during the data acquisition. The number of scans was 256–4000 to reach an acceptable signal-to-noise ratio. The ^13^C scale is referenced to crystalline γ-glycine (176.03 ppm for ^13^C). Considering that the cross-polarization efficiency for a given CP contact time is different for different functional groups, the presented quantitative analysis is subject to a certain error (uncertainty). The test based on the comparison of the routinely recorded ^13^C CP/MAS NMR spectrum with the spectrum recorded with a single-pulse excitation (duration of the ^13^C 90deg pulse was 3 μs) and a very long repetition delay (60 s) showed that this experimental error is about ±5–6%. A PerkinElmer FTIR spectrometer (model Frontier) was used for measurements of FTIR spectra. The spectra (average of 32 scans, resolution 4 cm^–1^) were recorded using attenuated total reflection (ATR) in the range of 400–4000 cm^–1^. Scanning electron microscopy images (SEM) were performed on a JWS-7515, JEOL Ltd. scanning electron microscope. The samples were attached to a carbon tab for better conductivity, and afterward, a thin layer of Pt was sputtered on a sample’s surface prior to scanning analysis (for SEM investigations). SEM micrographs were taken at a magnification of 5000 times, at a 7 mm working distance, and 20 kV voltage applied. The adsorption and desorption isotherms of N_2_ were obtained at −196 °C using a Micromeritics TriStar II 3020 instrument. Prior to measurements, the samples were degassed under N_2_ stream (purity 6.0) using a programmed bilevel heating, with the first heating stage at 90 °C for 60 min, followed by the second heating stage at 110 °C for 240 min. The specific surface area of the samples was calculated by applying the BET theory to the nitrogen adsorption data within the 0.06–0.30 *p*/*p*_0_ range. The polyHIPE densities (ρ_PH_) were determined gravimetrically and then the porosities were calculated assuming a polymer (skeletal) density (ρ_P_) of 1.13 g·cm^–3^ according to the following equation:
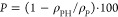


UV–vis DR spectroscopy was performed on a PerkinElmer Lambda 35 UV–vis spectrophotometer equipped with the RSA-PE-19 M Praying Mantis accessory for powdered samples in order to record the UV–vis diffuse reflectance spectra of the prepared materials. The background correction was performed with a white reflectance standard Spectralon^©^ (range of 200–900 nm). The optical band gap energies were determined using the Kubelka–Munk theory and Tauc plot as described by Macyk et al.^50^

## Results and Discussion

### Synthesis of Polyacetylene-Based PolyHIPE Networks

**Figure 1:**
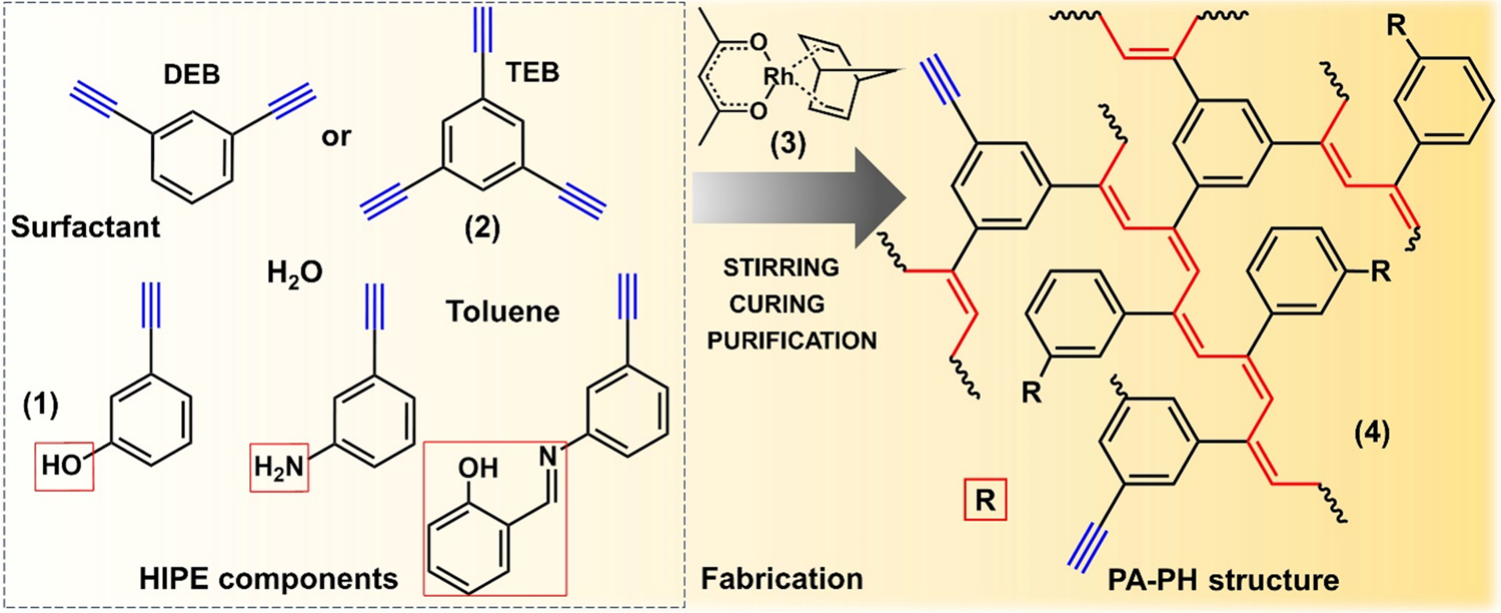
Schematic illustrations of the synthesis of PA–PH networks.

Building on our previous work incorporating 1,3-diethynylbenzene into a conjugated polyHIPE network,^22^ we set out to synthesize a library of polyacetylene-based polyHIPEs (PA–PH). 1,3-Di- and 1,3,5-triethynylbenzene monomers were homopolymerized to form nonfunctionalized PA–PH networks, abbreviated as PA-DEB and PA-TEB, respectively, while 3-ethynylphenol, 3-ethynylaniline, or *N*-salicylidene(3-ethynylaniline) monomers were copolymerized with two equivalents of 1,3,5-triethynylbenzene and formed networks, abbreviated as PA-OH, PA-NH_2_, and PA-SAL, respectively (Figure 1). The chain-growth coordination polymerization was used as polymerization chemistry with the mononuclear Rh(I) complex as the initiator. The networks produced consisted of π-conjugated PA chains hyper-cross-linked by benzenediyl and benzenetriyl links. To optimize the emulsion polymerization using the Rh(I)-catalyzed polymerization chemistry, a series of experimental parameters were investigated, such as the toluene–water phase ratio (set to ∼0.80), concentrations of the (co)monomers (1.7 mol·dm^–3^), initiator loading the (total concentration of the initiator differed according to the average number of ethynyl groups per (co)monomer molecule, so in all cases, 0.03 mol of initiator per mole of ethynyl groups were used), and the amount of surfactant (15 wt % according to the continuous phase of HIPE). The polymerization between (**1**) and (**2**) in the external (monomeric) phase took place immediately after the addition of (**3**) to the HIPE and resulted in a polyacetylene-based PH network (**4**) (Figure 1). In all cases, gelation was very rapid, with the HIPEs stopped to flow within minutes, even at room temperature. Due to the rapid gelation, the catalyst solution was only added at the end of the emulsion preparation, which gave us a few extra minutes to homogenize the HIPE and transfer it into a suitable mold. Final curing at 75 °C and subsequent purification/drying resulted in a brown, lightweight, and monolithic PA–PH. The gel formation was additionally confirmed by immersing the selected samples in liquid nitrogen when a point of apparent gelation had reached (the HIPEs stopped flowing) and then adding a large amount of dichloromethane. The samples did not dissolve, clearly indicating the formation of a chemically cross-linked gel. The polymerization yields were evaluated by setting the mass of dried monoliths relative to the mass of monomers and all PA–PH networks were prepared in quantitative yields, indicating a highly efficient Rh(I)-initiated polymerization in a two-phase HIPE system (Table 1).

**Table 1:** Characterization Data of Polyacetylene-Based PolyHIPEs

sample	PA-OH	PA-NH_2_	PA-SAL	PA-TEB	PA-DEB
*P* [%]^a^	95	96	95	97	96
ρ_PH_ [g·cm^–3^]^b^	0.05	0.04	0.05	0.03	0.04
*d*_V_ [μm]^c^	24 ± 5	23 ± 4	16 ± 3	24 ± 3	15 ± 2
*S*_BET_ [m^2^·g^–1^]^d^	471	308	273	1055	449
*V*_mic_ [cm^3^·g^–1^]^e^	0.18	0.12	0.11	0.41	0.18

a Porosity.

b PH density.

c Av. void size estimated from SEM images.

d Specific surface area.

e Volume of micropores determined from the N_2_ physisorption analysis.

### Molecular and Porous Structure

**Figure 2:**
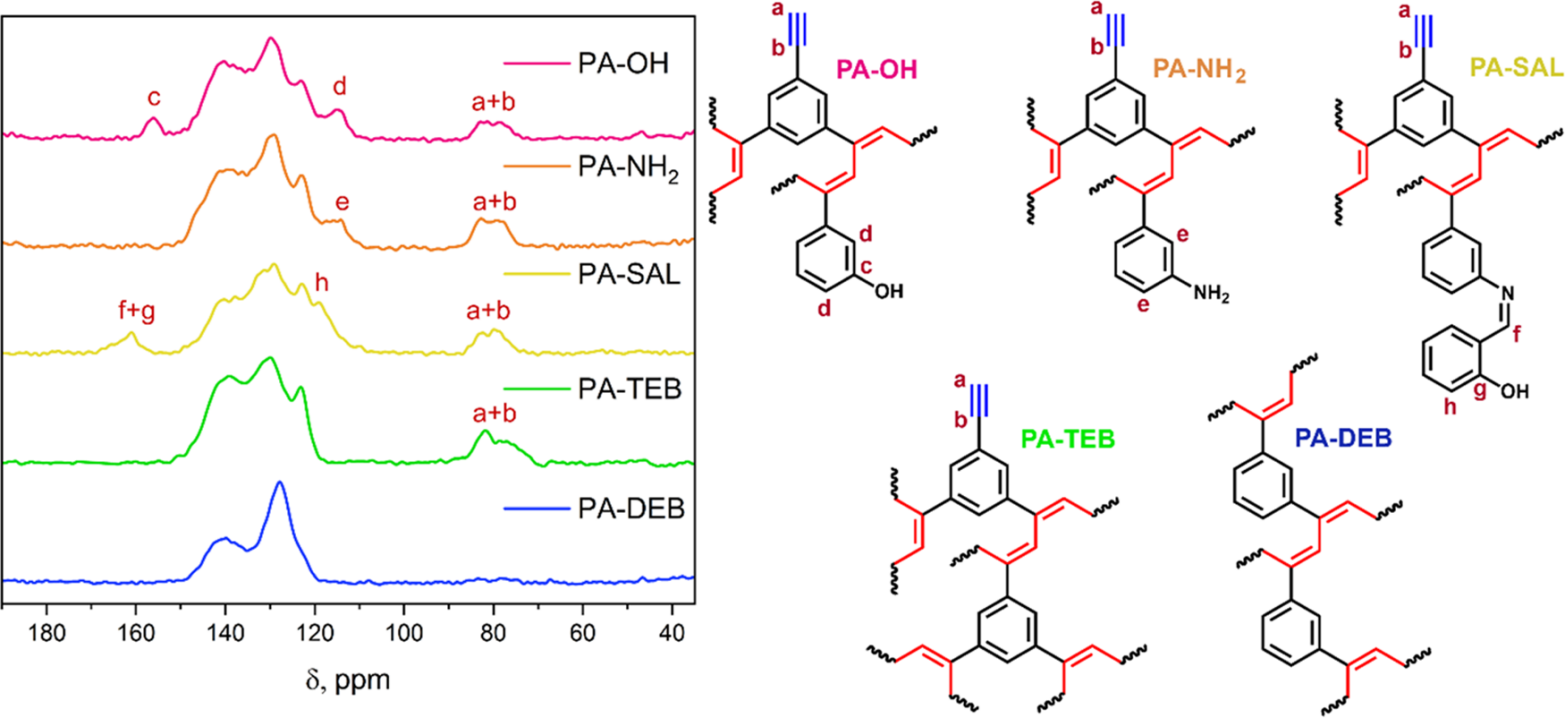
^13^C CP/MAS NMR spectra of prepared PA–PH networks.

As evidenced by FTIR and ^13^C CP/MAS NMR analyses, all PA–PHs consist of a typical arene-linked polyacetylene network motif. The FTIR spectra (Figure S1) show the bands associated with the benzene cross-linking and side units and the ethenylene units of the main chains in the range of 500–900 cm^–1^ and 1500–1600 cm^–1^, respectively. The presence of a certain amount of unconverted ethynyl side groups in the PA–PH networks was clearly confirmed by the bands at around 3300 cm^–1^ (and 2110 cm^–1^). ^13^C CP/MAS NMR spectra of all PA–PH networks together with their structures are shown in Figure 2. All networks showed broad, partially resolved signals in the region 115–150 ppm corresponding to aromatic carbons and carbons of the polyene main chain. The ^13^C CP/MAS NMR further confirmed the presence of substituents (−OH, –NH_2_, −CH=N–) attached to the benzene rings in copolymer networks by the characteristic signals of the aromatic carbon atoms in the vicinity of these groups: in PA-OH spectrum at 156 ppm (C_Ar_–OH) and 115 ppm (C_Ar_-C_Ar_–OH), in PA-NH_2_ spectrum shoulder at 146 ppm (C_Ar_–NH_2_) and 114 ppm (C_Ar_-C_Ar_-NH_2_) and in PA-SAL spectrum at 161 ppm (−HC=N–C_Ar_) and 119 ppm (C_Ar_-C_Ar_–OH). In the ^13^C CP/MAS NMR spectra of all networks obtained with TEB, a signal at about 83 and 76 ppm is clearly visible, which is due to the unreacted ethynyl carbon atoms. The average content was estimated to be 0.8 unreacted ethynyl groups per 1,3,5-triethynylbenzene monomer unit in PA-TEB. In contrast, almost complete conversion of ethynyl groups was observed in PA-DEB (Figure 2). In both systems, a new signal appears at δ = 140 ppm, which can be attributed to the carbons in the polyene backbone (Figure S2).

**Figure 3:**
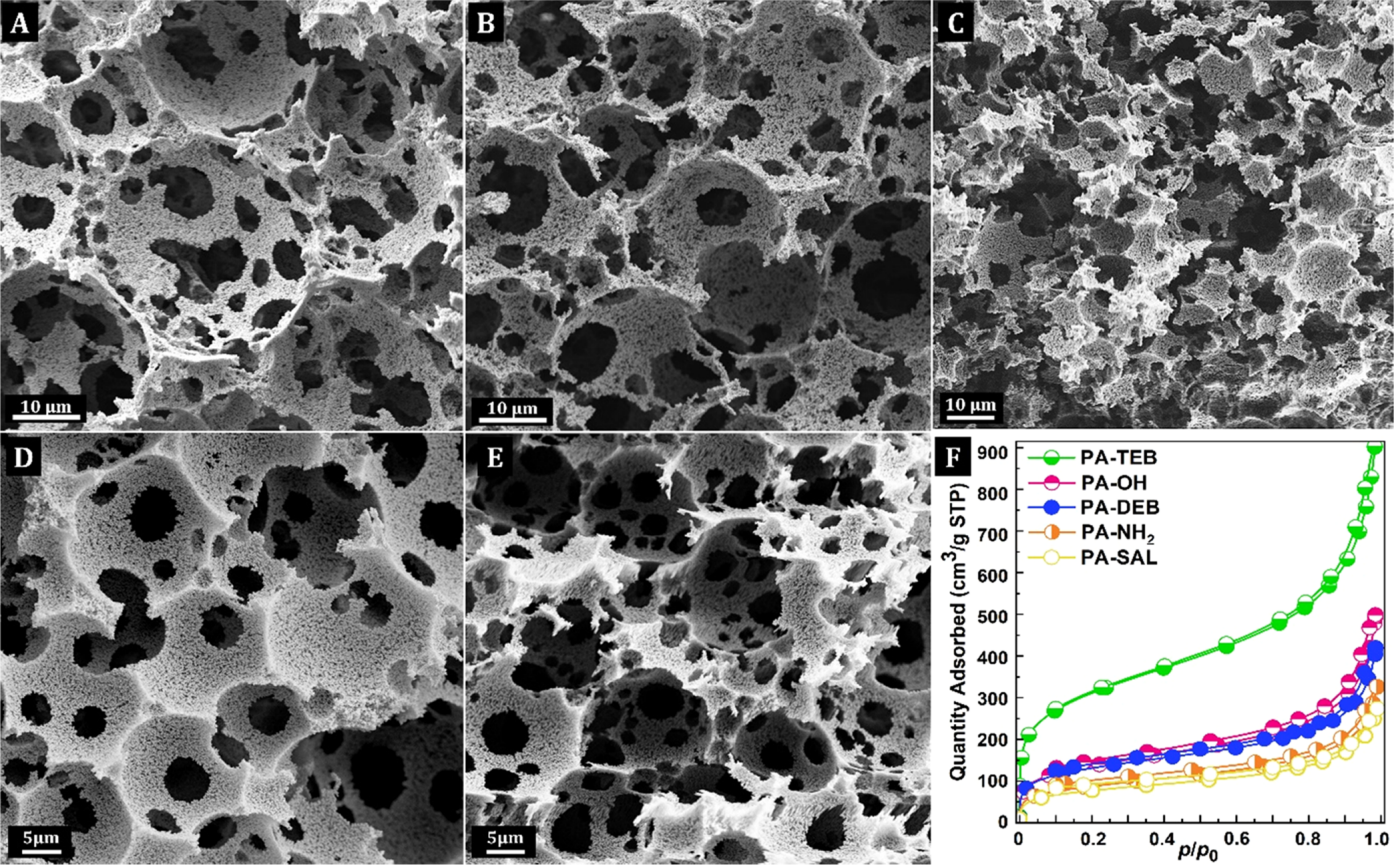
Porous structures (SEM) of (A) PA-OH; (B) PA-NH_2_; (C) PA-SAL; (D) PA-TEB; (E) PA-DEB; and (F) N_2_ adsorption–desorption isotherms of PA–PHs.

The porous structures typical of PHs are shown in Figure 3. Analysis by SEM revealed that the HIPE structure was templated within the PA–PHs and all had a 3D-interconnected microcellular morphology with an average void size between 15 ± 2 and 24 ± 5 μm, respectively. SEM analysis further revealed small macropores with diameters of about 200 nm within the polymer matrix, forming a hierarchical porous system (Figure S3). The PH densities (ρ_PH_) were relatively low and varied between 0.03 and 0.05 g·cm^–3^, suggesting the presence of even smaller pores, i.e., in the meso- or microlength scale (vide infra) (Table 1). The total porosities (*P*) of the PA–PHs were calculated from the ρ_PH_ by assuming a skeletal density (ρ_P_) of 1.13 g·cm^–3^ for trans-PA^51^ and were surprisingly high for 80% of the internal phase content, i.e., ≥95% (Table 1). The porous properties and associated specific surface areas (*S*_BET_) were further analyzed using nitrogen adsorption–desorption measurements. All PA–PHs showed a typical type II isotherm with a steep increase at *p*/*p*_0_ ≈ 1 due to the presence of macropores, and an additional increase in N_2_ uptake up to 0.1 *p*/*p*_0_, indicating the presence of micropores. The volume of the micropores was determined between 0.11 and 0.41 cm^3^·g^–1^ (derived from the N_2_ isotherms; Table 1) and, as expected, reflected in the *S*_BET_. The highly cross-linked PA-TEB network, which had the highest micropore volume (0.41 cm^3^·g^–1^), exhibited *S*_BET_ of 1055 m^2^·g^–1^ while approximately half *S*_BET_ (449 m^2^·g^–1^) and half micropore volume (0.18 cm^3^·g^–1^) was found for PA-DEB. Functionalized copolymer networks PA-OH, PA-NH_2_, and PA-SAL revealed *S*_BET_ of up to 471 m^2^·g^–1^ with micropore volumes between 0.11 and 0.18 cm^3^·g^–1^ (Figure 3F).

### Removal of Bisphenol A (BPA) by Adsorption and Photooxidation

**Figure 4:**
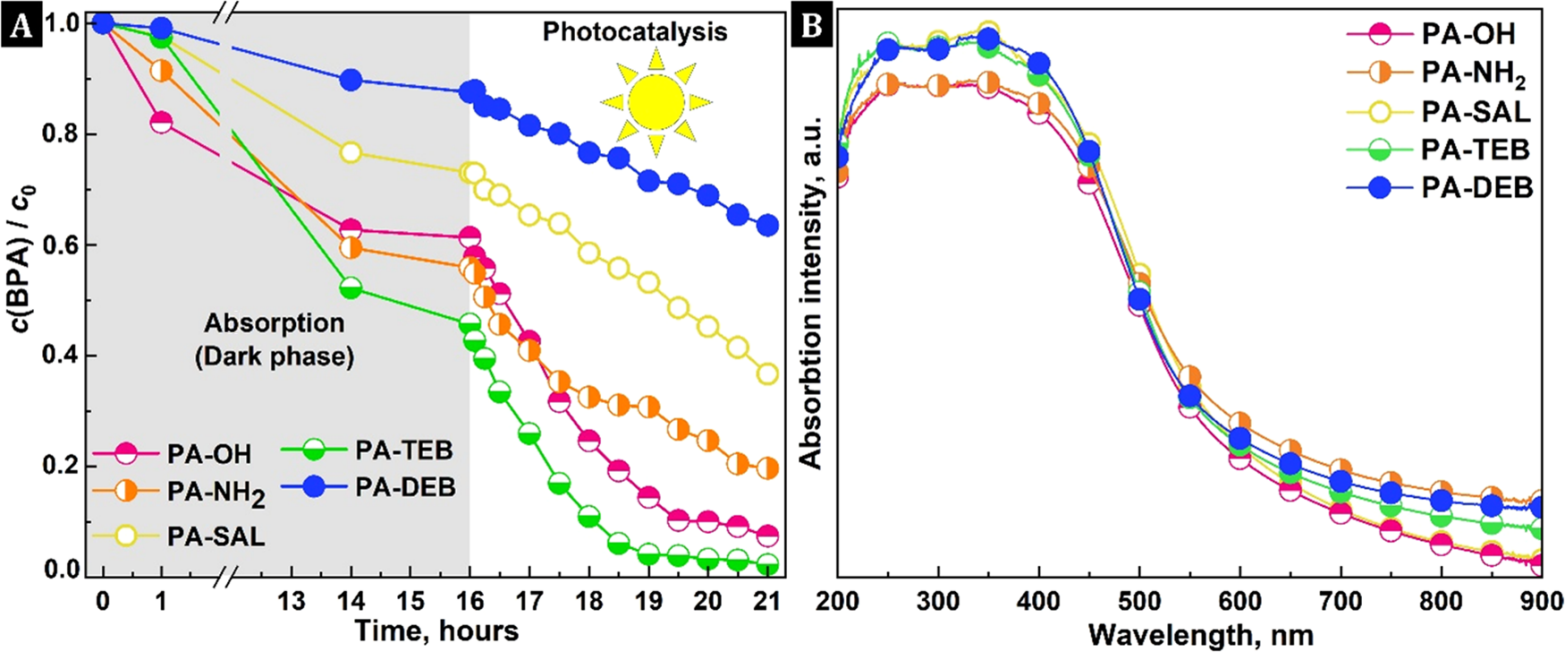
(A) Adsorption and visible-light-driven photooxidation of water-dissolved BPA and (B) UV–vis DRS analysis of the PA–PHs.

The high surface area and the π-conjugated nature of the polyacetylene networks enable PA–PHs to function as an adsorptive photocatalyst, i.e., simultaneously as an adsorbent and photocatalyst (Figure S4). To investigate the adsorption performance of PA–PHs, we first carried out an adsorption test in which the BPA concentrations in the supernatant were measured at different time intervals, as shown in Figure 4A (dark adsorption phase). The removal of BPA for PA-DEB, PA-TEB, PA-SAL, PA-NH_2_, and PA-OH reached about 1, 2, 3, 9, and 18% within the first hour and 11, 48, 24, 41, and 38% within 14 h when we are slowly reaching the adsorption plateau (except for PA-TEB which probably requires more time for adsorption equilibrium). Adsorption tests suggest that the specific surface area, π-conjugation, and the functional groups on the pore surface affect the amount of adsorbed BPA. The first rationale for the BPA adsorption on the surface of PA-DEB and PA-TEB, which is particularly high in the case of PA-TEB, is as follows. In addition to the strong hydrophobic effect, that often drives the adsorption of pure hydrocarbon networks, the high π-electron polarizability at the surfaces, similar to the PA network, has also proven to be an important factor in the adsorption of aromatic organic pollutants. In the literature, this phenomenon is known as π–π electron donor–acceptor (EDA) interaction^52−55^ and is based on the interaction between the π-electron-poor regions (considered as π-acceptors) and electron-rich aromatic organic molecules (considered as π-donors). The high adsorption of BPA on the surface of PA-TEB is therefore mostly due to the π–π electron coupling between the π-electron-rich region in the PA network and the electron-poor benzene rings of BPA. On the other hand, adsorption in the case of PA-OH, PA-NH_2_, and PA-SAL is driven by other mechanisms. It is interesting that PA-OH (471 m^2^·g^–1^) and PA-NH_2_ (308 m^2^·g^–1^) have a similar *S*_BET_ to the PA-DEB network (449 m^2^·g^–1^), but their adsorption capacities are similar to those of PA-TEB. Apparently, the N- and O-heteroatoms in the PA-NH_2_ and PA-OH networks function as active sites on the surface that enable efficient BPA removal by combining π–π electron coupling with the (weak) hydrogen bonding interaction mechanism.^48,56−59^

Next, PA–PHs were investigated as heterogeneous photocatalysts for visible-light-driven photooxidation of BPA dissolved in water. First, UV–vis diffuse reflectance spectroscopy was used to study the light-harvesting ability of PA–PHs and revealed that all are visible-light-active materials. The UV–vis DRS spectra display a broad band with a maximum of around 350 nm and corresponding optical absorption band edges between 612 and 630 nm (Figure 4B). The band gaps were determined through Kubelka–Munk transformed reflectance spectra. The Tauc plots depicted in Figure S5 indicate that the optical band gaps are in the range of 2.2–2.3 eV. The photocatalytic activities of the PA–PH networks in the BPA photooxidation were then investigated as shown in Figure 4A. A typical photocatalysis experiment was performed with the addition of 12.5 mg of a network as a catalyst to 100 mL of aqueous BPA solution (*c*_BPA_ = 10 mg·dm^–3^). In the first step (dark adsorption phase), only the adsorption of BPA on the catalyst surface took place, followed by the photocatalysis phase, in which the reaction system was illuminated with visible light (λ > 420 nm). As shown in Figure 4A, PA-DEB, PA-TEB, PA-SAL, PA-NH_2_, and PA-OH showed activity in which 24, 43, 37, 38, and 58% of BPA was removed by oxidation in 5 h of illumination, respectively (decrease of the BPA concentration during the light phase). Since hydroxyl radicals (OH^•^) are the most reactive oxidizing species in the degradation of organic pollutants, their formation was monitored during the photoexcitation of PA–PHs in the presence of the fluorescent probe molecule coumarin (COUM). COUM reacts with OH^•^ radicals to form 7-hydroxycoumarin (7-OHC); therefore, the fluorescence intensity of 7-OHC can be related to the amount of OH^•^ radicals produced by a given catalyst sample.^60^ The results of the coumarin oxidation experiments performed by illuminating the PA-OH and PA-NH_2_ networks with visible light are shown in Figure S6. The results confirm that both networks can generate OH^•^ radicals under visible-light illumination. Finally, two control experiments were performed, namely, COUM oxidation in the absence of the polyHIPE photocatalyst and the stability of the polyHIPE photocatalyst under photooxidation conditions (see the Experimental Section). The control experiment without PA-OH and PA-NH_2_ photocatalyst showed no photooxidation of COUM to 7-OHC, while the PA-TEB network exhibited high chemical robustness, as HPLC and ^13^C CP/MAS NMR analyses confirmed that neither segments were leached from the network into the water, nor the covalent structure was altered under photooxidation conditions.

Considering both the adsorption and photooxidation activity (Figure 4), all PA–PHs are excellent adsorptive photocatalysts. The adsorption of BPA on the pore surface had no effect on the further photocatalytic efficiency, and in the case of PA-TEB, we even succeeded in completely removing BPA from the aqueous solution.

## Conclusions

In summary, a series of polyacetylene-based polyHIPEs were prepared by insertion coordination polymerization of HIPE templates. All were hierarchically porous polymers with micropore volumes between 0.11 and 0.41 cm^3^·g^–1^ and exhibited high *S*_BET_ values (273–1055 m^2^·g^–1^). All PA–PHs also exhibit significant semiconducting properties, such as a strong light-harvesting ability in the visible-light region with optical band gaps in the range of 2.20–2.33 eV. The porosity and electronic properties can be adjusted by selecting suitable building blocks. The PA–PHs were then used to remove BPA from water and achieved near-quantitative adsorption/photooxidation efficiency. The PA-TEB, a pure hydrocarbon network, with the highest *S*_BET_ (1055 m^2^·g^–1^) showed the highest BPA adsorption activity. On the other hand, PA-OH and PA-NH_2_ networks with significantly lower *S*_BET_ (471 and 308 m^2^·g^–1^, respectively) revealed similar adsorption capacities, which can be attributed to the polar functional groups on the pore surface. Importantly, this high adsorption capacity did not affect the subsequent photocatalytic activity. All PA–PHs were also used effectively for BPA photooxidation in water, with between 24 and 58% of BPA being successfully degraded. Due to their highly porous and π-electron-delocalized polyacetylene network structure, PA–PHs have therefore been successfully used both as an efficient adsorbent for BPA and as a heterogeneous photocatalyst for its degradation.

## Supporting Information Available

Procedures and details about characterization and description of adsorption/photooxidation (PDF)
