# Supplementary material for: Hierarchically Porous Polyacetylene Networks: Adsorptive Photocatalysts for Efficient Bisphenol A Removal from Water
Source: ACS Polym Au. 2024 Jun 6;4(5):420–7. doi: 10.1021/acspolymersau.4c00032 (PMC11468696; doi:10.1021/acspolymersau.4c00032)
Supplement: Supplementary file 1 — lg4c00032_si_001.pdf [file lg4c00032_si_001.pdf]

Supporting information for paper:

Hierarchically Porous Polyacetylene Networks: Adsorptive Photocatalysts for Efficient Bisphenol A Removal from Water

*David Šorm[a], Jiří Brus[b], Albin Pintar[c], Jan Sedláček[a] and Sebastijan Kovačič[c,d]**

[a)] Department of Physical and Macromolecular Chemistry, Faculty of Science, Charles University, Hlavova 2030, Prague 2, 128 43, Czech Republic

[b)] Institute of Macromolecular Chemistry, Czech Academy of Sciences, Heyrovský Sq. 2, 162 00, Prague, Czech Republic

[c)] Department of Inorganic Chemistry and Technology, National Institute of Chemistry, Hajdrihova 19, SI-1001 Ljubljana, Slovenia

[d)] University of Maribor, Faculty of Chemistry and Chemical Engineering, Smetanova 17, SI-2000 Maribor, Slovenia; E-mail: sebastijan.kovacic@um.si

FTIR characterization

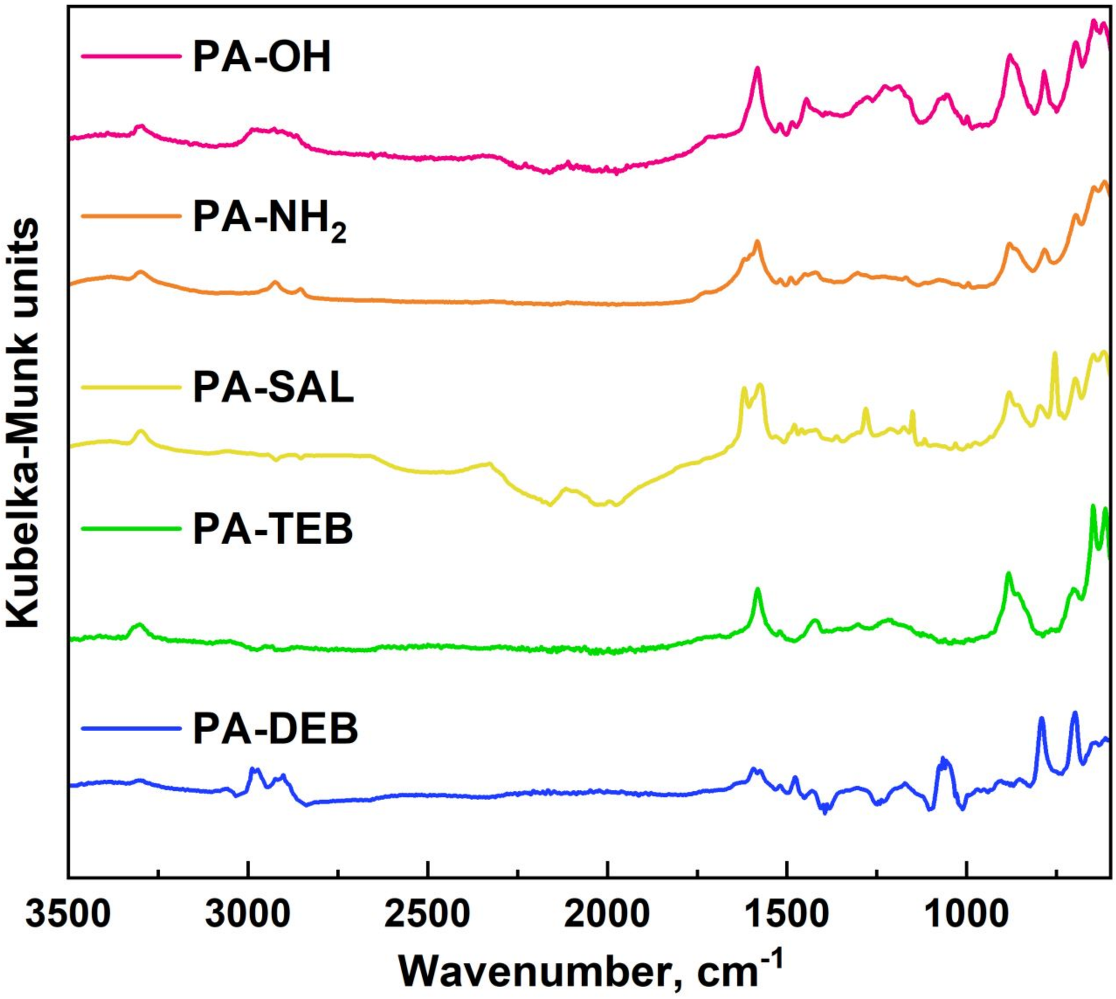

Figure S1. FTIR spectra of PA-PHs: PA-OH, PA-NH_2, PA-SAL, PA-TEB and PA-DEB.

^{13}C CP/MAS characterization

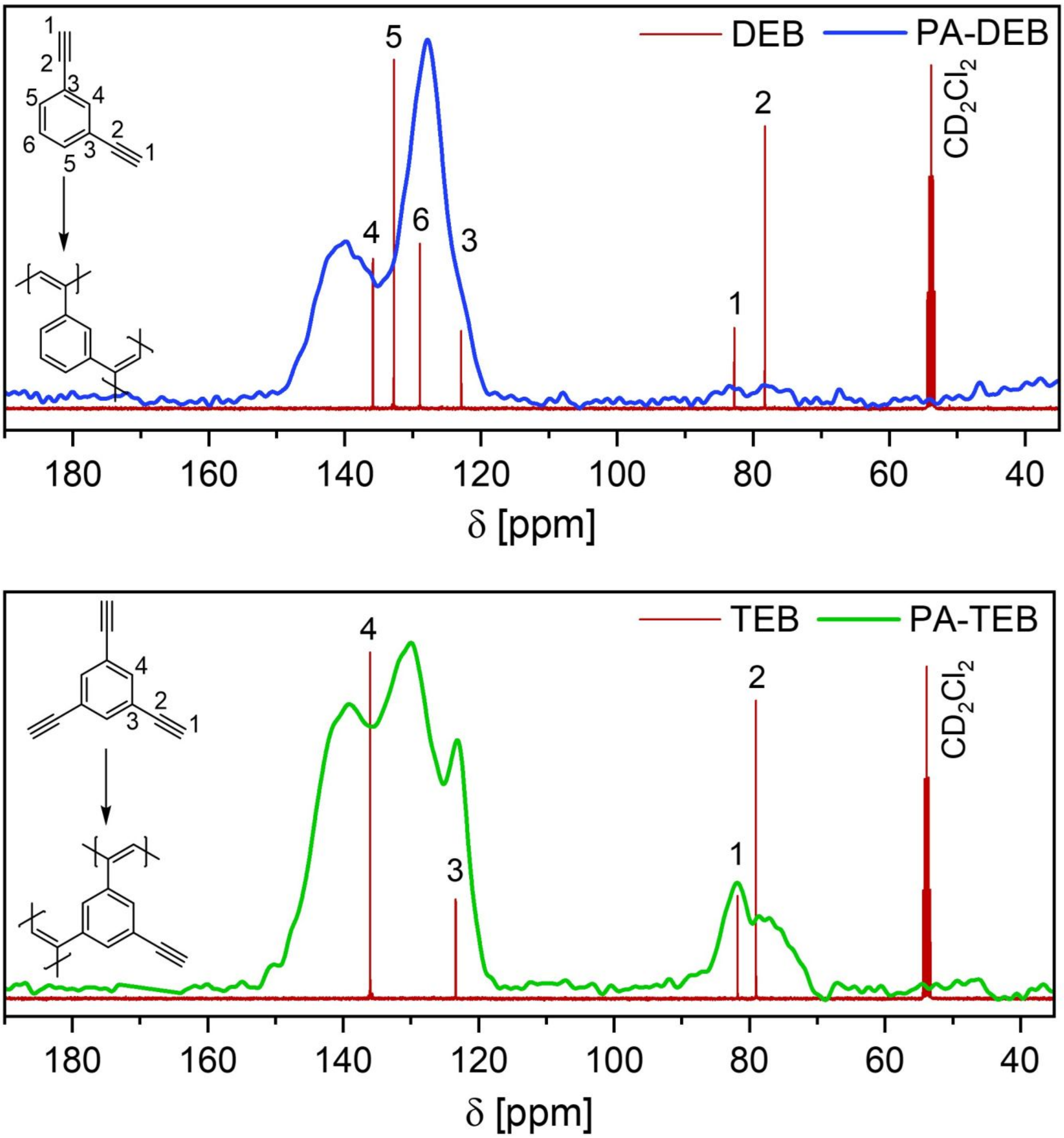

Figure S2. ^{13}C CP/MAS NMR spectra of homopolymers PA-DEB and PA-TEB compared with ^{13}C NMR spectra of respective monomers 1,3-diethynylbenzene and 1,3,5-triethynylbenzene measured in CD_2Cl_2.

High magnification SEM analysis

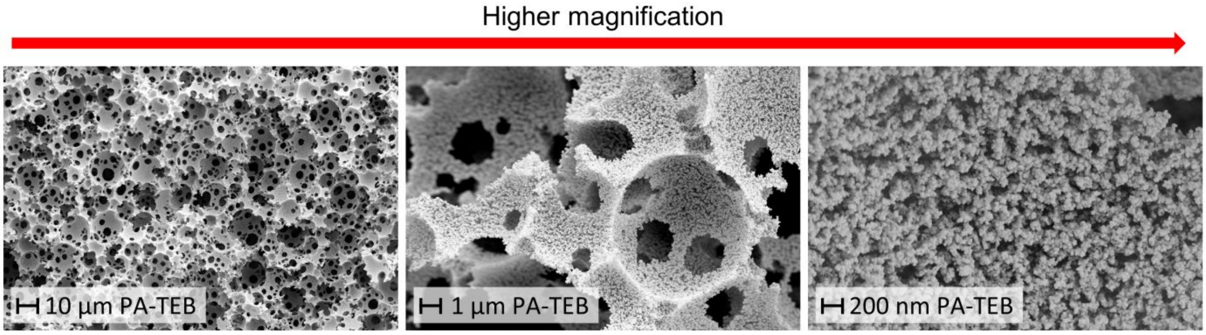

Figure S3. Comparison of different magnification by SEM on network PA-TEB.

Adsorption-&-photodegradation of bisphenol A (BPA) on the PA-PH

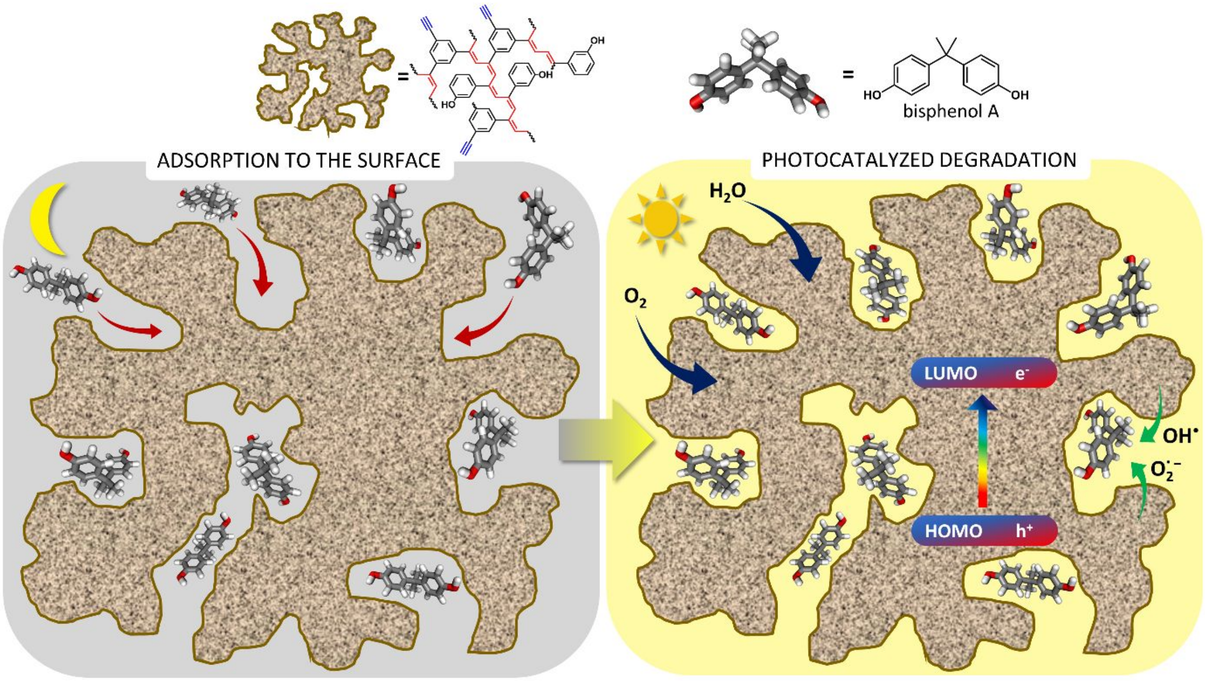

Figure S4. Adsorption of bisphenol A and photocatalytic processes on the surface of polyacetylene-based polyHIPE network.

UV VIS DRS characterization

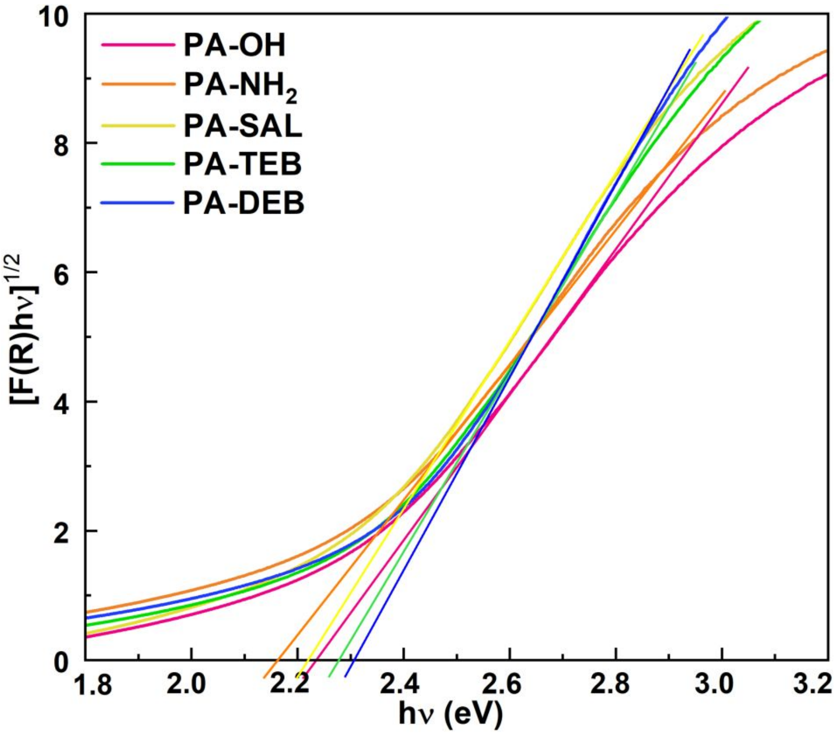

Figure S5. The Tauc plots.

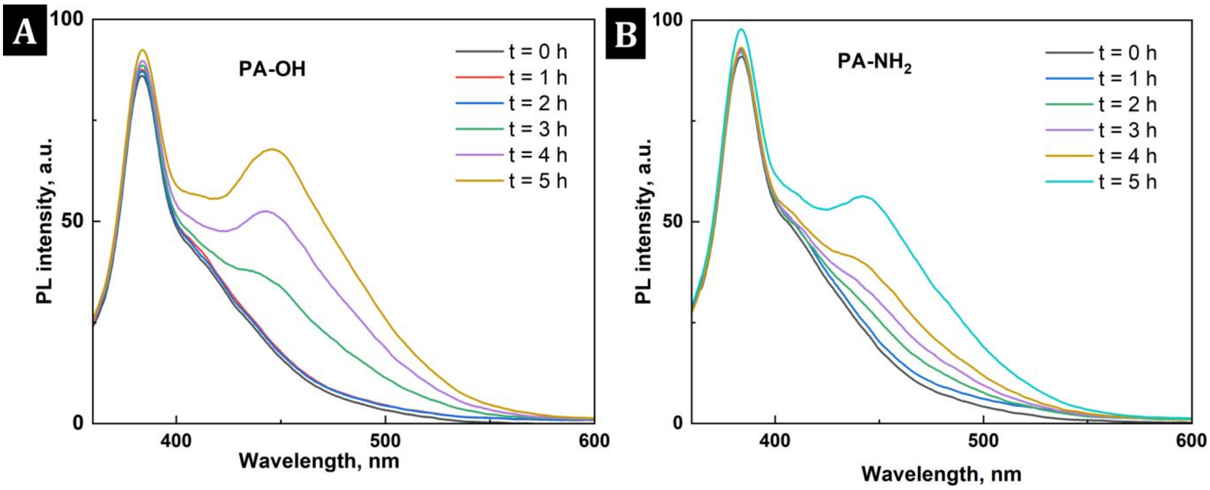

Figure S6. Coumarin test for the observation of 7-hydroxycoumarin generation using PA-OH (**A**) and PA-NH_2 (**B**) networks. Maximum of coumarin @ λ=384 nm; maximum of 7-hydroxycoumarin @ λ=445 nm.
